# Effect of anterior capsule polish on visual function: A meta-analysis

**DOI:** 10.1371/journal.pone.0210205

**Published:** 2019-01-08

**Authors:** Meng-yao Han, Ai-hua Yu, Jing Yuan, Xiao-jun Cai, Jiang-bo Ren

**Affiliations:** Department of Ophthalmology, Zhongnan Hospital of Wuhan University, Wuhan, Hubei, China; Faculty of Medicine, Cairo University, EGYPT

## Abstract

**Purpose:**

To investigate the relationship between anterior capsule polish and visual function.

**Methods:**

Data were obtained from Pubmed, Embase, Web of Science, WanFang, VIP and CNKI up to the end of May 2018, without any date or language restrictions for trials. The modified Jadad scale and the newcastle-ottawa scale were used to assess the quality of included studies. Uncorrected visual acuity (UCVA) and posterior capsule opacification (PCO) were used as outcome variables. Data on anterior capsule polish were pooled using weighted, random-effect meta-analysis.

**Results:**

One randomized controlled trial and 4 observational cohort studies involving 2533 patients were included in the analyses. There was a statistically significant difference of UCVA (OR 1.92, 95% CI 1.41–2.61) between the polish group and the control group, indicating that anterior capsule polish improved UCVA. Further studies with continuous data also suggested that anterior capsule polish was associated with good UCVA (MD 0.11, 95% CI 0.06–0.16). Posterior capsule opacification rate for 1-year or longer follow-up were extracted for 2561 eyes in 3 studies. Posterior capsule opacification rate was lower in the anterior capsule polish group according to summary odds ratio on PCO rate (OR 0.42 95% CI 0.24–0.73).

**Conclusions:**

Anterior capsule polish prevents complication of modern cataract surgery and benefits on visual function in short term follow-up period.

## Background

Age-related cataract is lens opacification and the common cause of blindness for elders in the world. The Chinese Center for Disease Control and Prevention estimated that 2.91 million Chinese aged 50 years or older had cataract in one or both eyes in 2010.[1,2] With the rapid aggravation of aging population in China, the increased number of age-related cataract patients is predictable and palpable. Phacoemulsification is used to emulsify the cataract by ultra-sound energy, and intraocular lenses (IOLs) are then inserted into the bag.[3] Approximately 50% of adults and all the children have some form of posterior capsule opacification (PCO), which is caused by the remained lens epithelial cells (LECs) in the capsule bag and one of the most common complications after phacoemulsification.[4,5] Capsule opacification may lead to visual impairment including straylight.[6,7] The popular treatment of PCO is Nd:YAG laser capsulotomy; however, there are rare but significant complications of capsulotomy, such as raised intraocular pressure (IOP), intraocular lens pitting, intraocular lens cracks, cystoid macular oedema, retinal detachment and corneal burns.[8]

The incidence of PCO could be prevented by multiple methods, including anterior capsule polish, the sharp rim of IOLs, the material of IOLs, posterior capsulorhexis and medicine.[9–14] However, intraoperatively posterior capsulorhexis increases the risk of vitreous loss. While sharp-edged IOL forms a mechanical barrier, the remnant LECs result in capsule contraction and anterior capsule opacification. Capsule contraction lead to frontward motion of IOLs and reduction in the free optic zone. Therefore, it is important to decrease occurrence of capsule opacification and to improve capsular stability by removing LECs intraoperatively. LECs could be destroyed by several means, such as polish, 5-fluorouracil, and other medicine.[15] The side effect of medicine is the damage corneal endothelium and other ocular tissue, resulting in corneal opacification. Polish, as a kind of mechanical method, is thought to safely remove remnant LECs. Studies found that intraoperatively anterior capsule polish improved the axial stability of IOL.[16]

Although many studies have characterized the effectiveness of anterior capsule polish on PCO, there was little concentration on the effect of polish on visual function. In this study, we performed a meta-analysis to investigate the effect of anterior capsule polish on visual function.

## Methods

This systematic review and meta-analysis were performed in an academic medical setting according to the Preferred Reporting Items for Systematic Reviews and Meta-analyses (PRISMA) guidelines.

### Search strategy

The reports of anterior capsule polish were searches in consultation with Pubmed, Embase, Web of Science, WanFang, VIP, China National Knowledge Internet (CNKI) electronic databases for the reports of anterior capsule polishing up to the end of May 2018. No date or language restriction was used in the electronic searches for the trials. Neither was study design. “*Cataract*” and “*polishing*” were the main terms used for comprehensive literature search to identify relevant studies. A hand search by reading references of identified studies was performed by 2 authors (HMY and YJ) independently. The detailed searching strategy for Pubmed was listed as followed: ("cataract "[MeSH Terms] OR ("cataract"[All Fields]) AND ("anterior capsule"[MeSH Terms] OR "anterior capsule"[All Fields]) AND "polishing"[All Fields].

### Study selection

The eligibility of studies was assessed by 2 authors (HMY and YJ) independently via reading titles and abstracts. Endnote was used as the literature manager system to remove the duplicated publications. After that, 2 authors (HMY and YJ) independently screened the full-text papers with the inclusion criteria for the meta-analysis: (1) Participants, population of interest were patients with age-related cataract; (2) intervention, anterior capsule was polished intraoperatively; (3) outcome variables, at least 1 of primary outcome was reported: PCO rate, uncorrected visual acuity (UCVA).

### The assessment of the identified studies quality

The methodological quality of identified studies was assessed by 2 reviewers (HMY and YJ) independently to reduce the influence of different bias. The quality of randomized controlled trials (RCTs) was identified by the modified Jadad scale with a scale of 0 to 7. The modified Jadad scale included the following domains: randomization, blinding and patient attrition. Studies with scores higher than 4 were regarded as high quality.[17] The quality of cohort studies included in the review was assessed using the newcastle-ottawa scale, which included selection, comparability, and outcome. A third author (CXJ) was required in case of disagreements between 2 reviewers for the assessment of study quality.[18]

### Data extraction

The data were extracted from high quality studies by 2 authors (HMY and YJ) independently. The characteristics of eligible studies were recorded as first author, year of publication, study design, participants number, the period of follow-up, polish area, IOL type and outcome variables. We contacted the correspond authors for the missing information. The primary outcomes were recorded at different time points, such as one-month or one-year. Visual function variables including PCO rate and UCVA were extracted at different time points. If there were published studies on the same group of patients, more recent and complementary data were used in the meta-analysis.

### Data synthesis and assessment of heterogeneity

All statistical analyses were performed using RevMan 5.2 software (The Cochrane Collaboration, Oxford, England). Studies defined as the publication year were classified into different subgroups, depending on the follow-up periods. We pooled dichotomous data in odds ratio (OR) with 95% confidence intervals (CI), such as PCO rate and patient number of good uncorrected visual acuity. Continuous data were pooled using mean differences (MD) with 95% CI, such as visual acuity. Heterogeneity was analyzed through chisquare test. Heterogeneity was calculated by *I*^2^ and the chi-square statistic. *I*^2^<50% was considered to no heterogeneity among the included studies in a meta-analysis. We evaluated the pooled summary effect in random effect model. Publication bias was assessed using the funnel plot.

## Results

### Included studies

**Fig 1:**
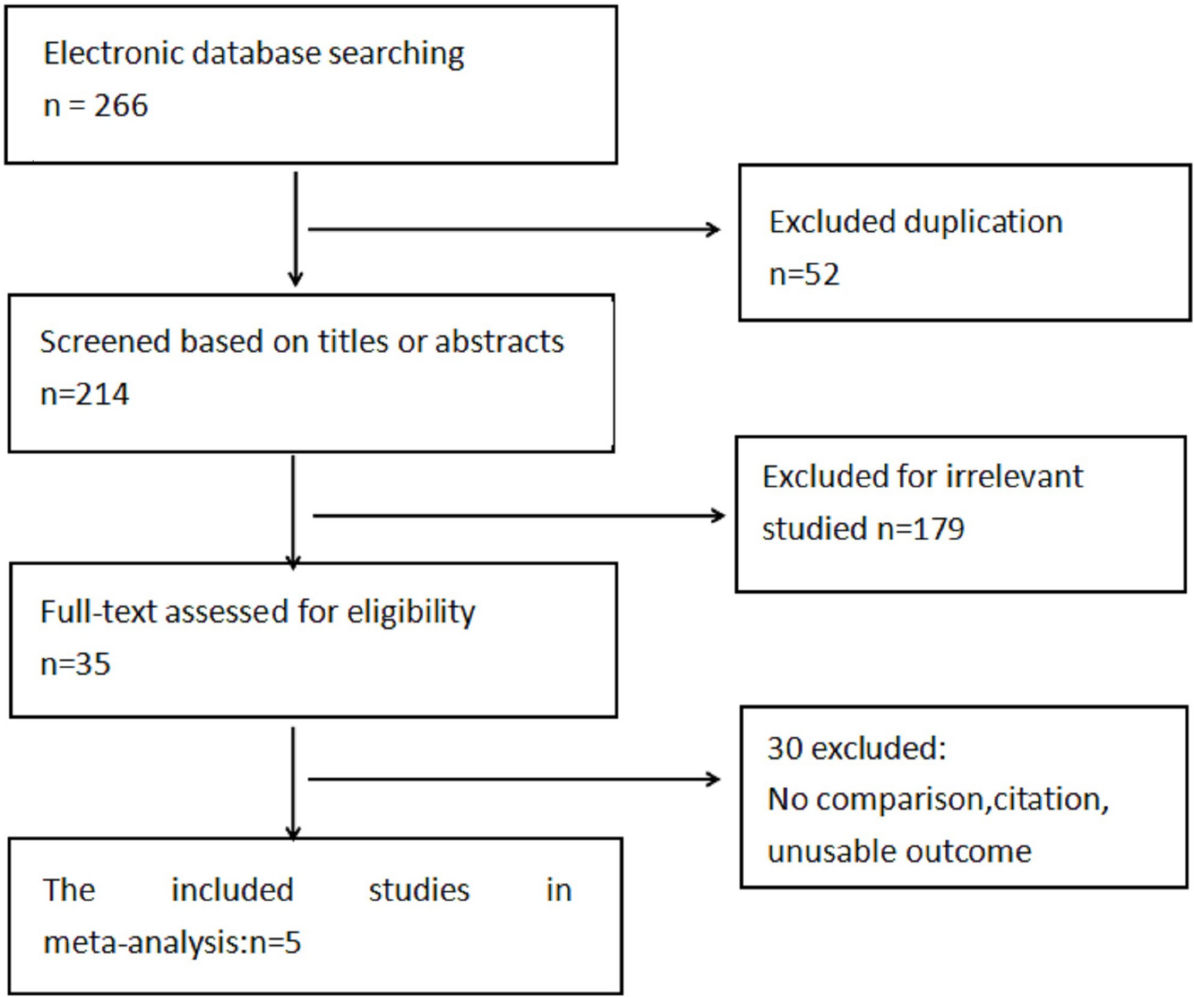
The flow chart of electronic database searching and screening.

Our search strategy identified 266 articles through electronic searches of multiple databases up date to May 2018. After deduplication, irrelevant articles were removed by screening titles and abstracts, excluding case reports, review and other objectives. A total of 35 full-text articles were selected for potential inclusion in the meta-analysis. Finally, 5 full-text articles were included in the meta-analysis with the inclusion criteria, of which 1 study identified RCT and the others were observational cohort studies. There were 3 Chinese studies and 2 English studies in the meta-analysis.[16,19–22] We also manually assessed the reference lists of all the retrieved original studies for potential information about anterior capsule polish intraoperatively. The flow chart of our search progress is shown in the Fig 1.

### Quality of articles and characteristics of selected articles

The characteristics of the included articles are shown in the Table 1. In total, there were 3 prospective studies and 2 retrospective studies in the meta-analysis, including 2713 eyes. The experiment group included 1373 eyes with anterior capsule polish. The follow-up periods were from 6 months to 3 years. The material of IOL had effect on visual function, therefore we paid additional attention on the types of IOL in the included studied. Hydrophilic acrylic IOL was applied in 2 studies and hydrophobic acrylate IOL was used in 1 study. The outcome variables of included studies were UCVA and PCO rate to evaluate visual function, and there were 4 studies included UCVA and 3 studies included PCO rate and 4 studies included UCVA, which was measured by logarithmic visual acuity chart. The methodological quality of included studies was presented in Tables 2 and 3. The modified Jadad scale was used to assess RCT, and the included RCT study with scores higher than 5 indicates good quality. The randomization of included RCT was performed in term of tossing coin. To address the accuracy of observational cohort studies, we adopted newcastle-ottawa scale to assess the quality of included cohort studies. The included cohort studies with 9 stars were considered high quality. The high quality of included cohort studies showed that the representative selection of participants and good comparability between experiment group and control group.[23] Therefore, 5 studies including 1 RCT and 4 cohort studies were deemed to be high quality in the meta-analysis.

**Table 1:** The characteristics of the included articles.

Author	year	study design	Participants (S/C)	follow-up(year)	polishing area	IOL type	outcome variables
Ma Yi	2001	retrospective	398(235/230)	1	anterior and equator capsule	-	VA, PCO rate
Ruhul.B	2012	retrospective	1907(1009/981)	3	anterior capsule	Hydrophilic acrylic,PCIOL	PCO rate
Da-qiang,Zhu	2012	prospective	106(53/53)	1	anterior capsule	Hydrophilic acrylic	VA, PCO rate
Gao	2015	prospective	40	0.5	anterior capsule	Hydrophobic acrylate	UCVA,ELP,ACD
Jia-li, Zhu	2017	prospective	56	1.5	anterior capsule	-	VA

**Table 2:** There is the newcastle-ottawa scale to assess the quality of included cohort studies. In newcastle-ottawa scale, studies with 9 stars were considered high quality.

Author	Year	Selection				Comparability		outcome		
Author	Year	1	2	3	4	polishing anterior capsule	other fator	Assessment of outcome	follow-up long enough	Adequacy of follow up
Ma Yi	2001	**✵**	**✵**	**✵**	**✵**	**✵**	**✵**	**✵**	**✵**	**✵**
Ruhul.B	2012	**✵**	**✵**	**✵**	**✵**	**✵**	**✵**	**✵**	**✵**	**✵**
Da-qiang,Zhu	2012	**✵**	**✵**	**✵**	**✵**	**✵**	**✵**	**✵**	**✵**	**✵**
Chen Jun-hong	2013	**✵**	**✵**	**✵**	**✵**	**✵**	**✵**	**✵**	**✵**	**✵**
Jia-li,Zhu	2017	**✵**	**✵**	**✵**	**✵**	**✵**	**✵**	**✵**	**✵**	**✵**

ACD: anterior chamber depth;ELP:effective lens position;PCO: posterior capsule opacification;UCVA:uncorrected visual acuity;VA: visual acuity;

✵: Srepresent one score of each item

**Table 3:** The modified Jadad scale to assess the quality of randomized controlled trials (RCTs).

Author	Randomization(2')	Concealment of allocation(2')	Double blinding(2')	Withdrawals and dropouts(1')	score(7')
Gao 2015	2	1	2	0	5

In modified Jadad scale, studies with scores higher than 5 were considered good quality

### Effects of polish on visual acuity

**Fig 2:**
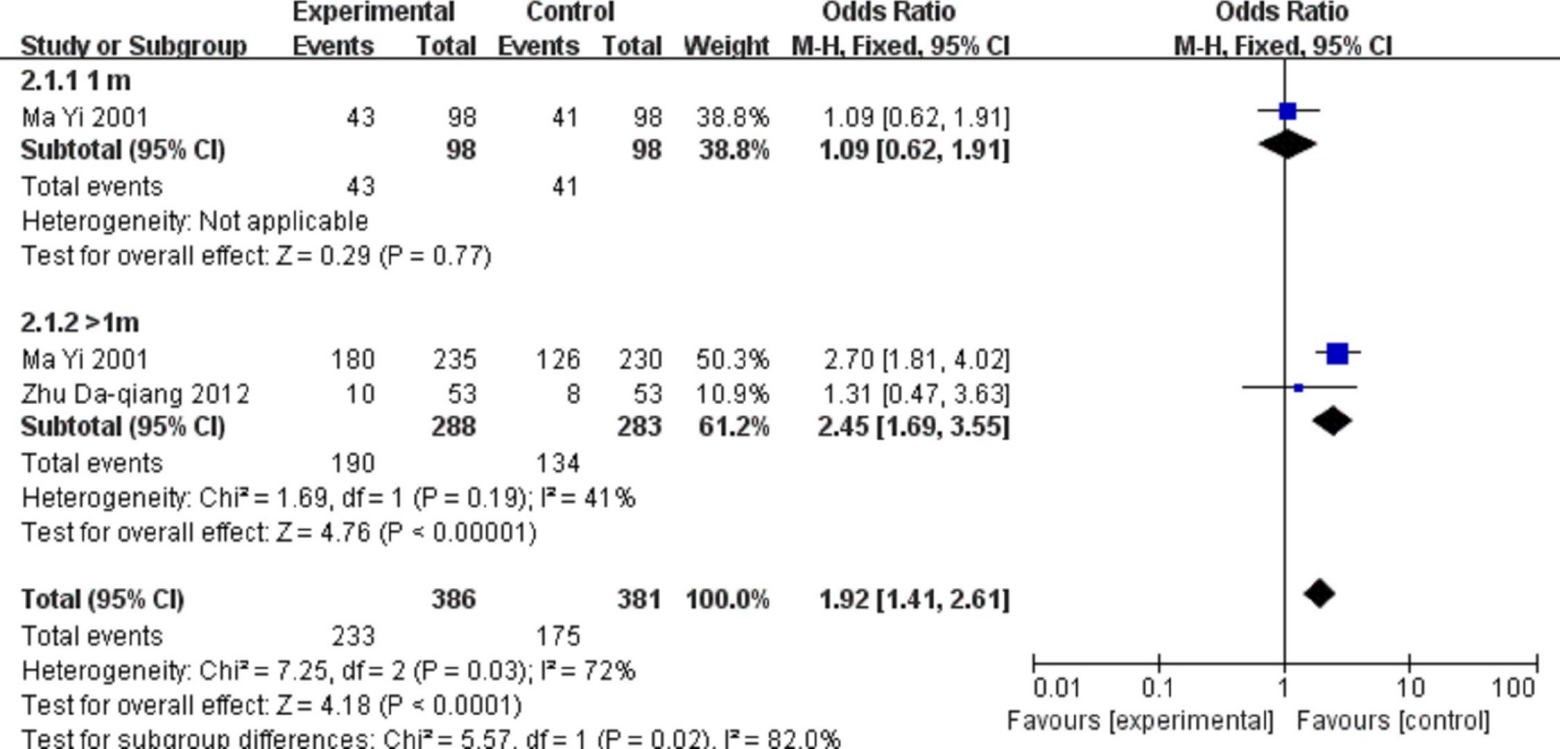
Patient number comparison of good uncorrected visual acuity between anterior capsule polish and unpolish groups after 1 month and longer follow-up. The invention of experiment group was anterior capsule polish; the control group was blank.

**Fig 3:**
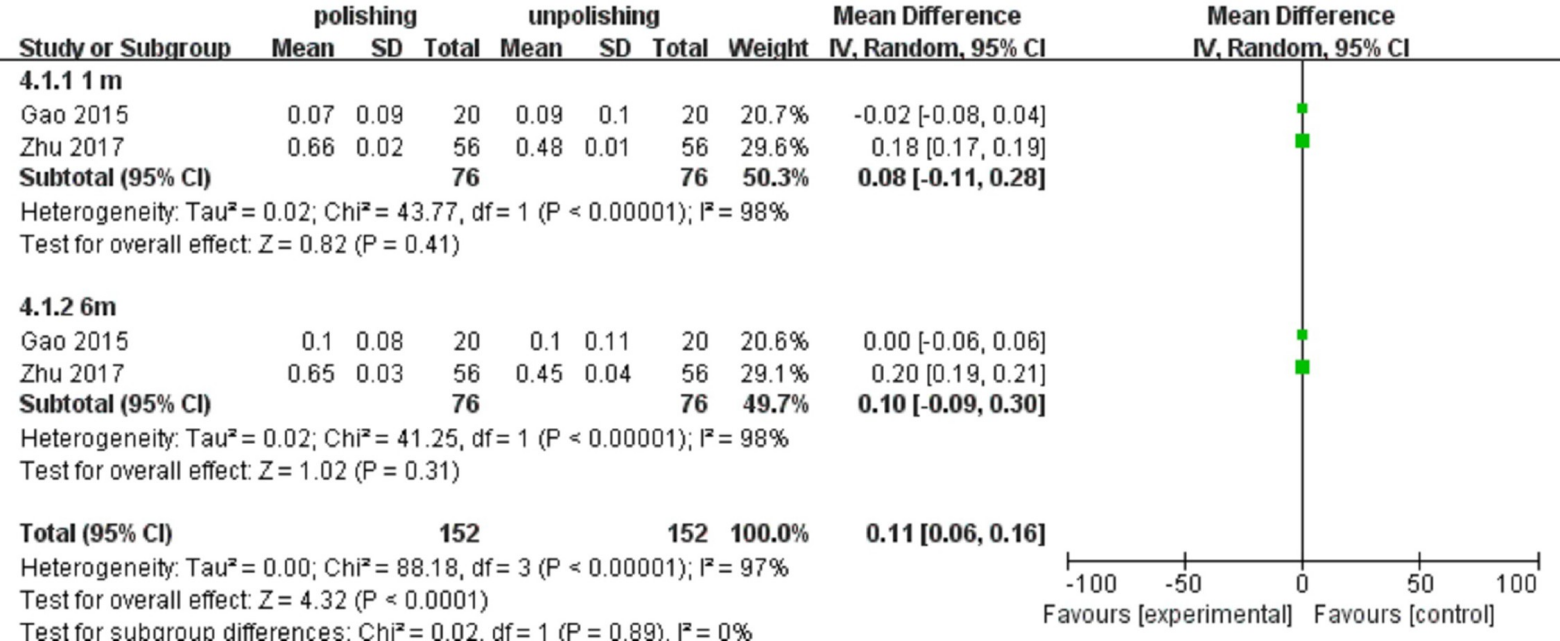
The continuous data of UCVA was compared between anterior capsule polish and unpolish groups after 1 month and 6 months follow-up. The invention of experiment group was anterior capsule polish; the control group was blank.

The meta-analysis aimed at effects of anterior capsule polish on visual function. The primary outcomes for the meta-analysis were UCVA and PCO rate. Uncorrected visual acuity as UCVA of more than 0.8 was defined as good. The number of patients with good UCVA was little at one time point, therefore we discussed UCVA in different subgroups at depended on different time points and pooled summary effects together. Our results showed that UCVA was improved by anterior capsule polish. The meta-analysis results comparing patient numbers of good UCVA between anterior capsule polish group and unpolish group were shown in Fig 2. There was no statistical heterogeneity between the studies (heterogeneity *I*^2^ = 0%). Based on 2 studies that evaluated uncorrected visual acuity after anterior capsule polish, there are more events in the polish group than unpolish group at both one-month (odds ratio 1.09, 95%CI 0.62 to 1.91) and longer follow-up period time points (odds ratio 2.45, 95%CI 1.69 to 3.55).[21,22] The summary effects also indicated that anterior capsule polish improved UCVA (odds ratio 1.92, 95%CI 1.41 to 2.61). Continuous data indicated that good UCVA was associated with anterior capsule polish (MD 0.11, 95%CI 0.06–0.16), which was shown in Fig 3.[16,19]

**Fig 4:**
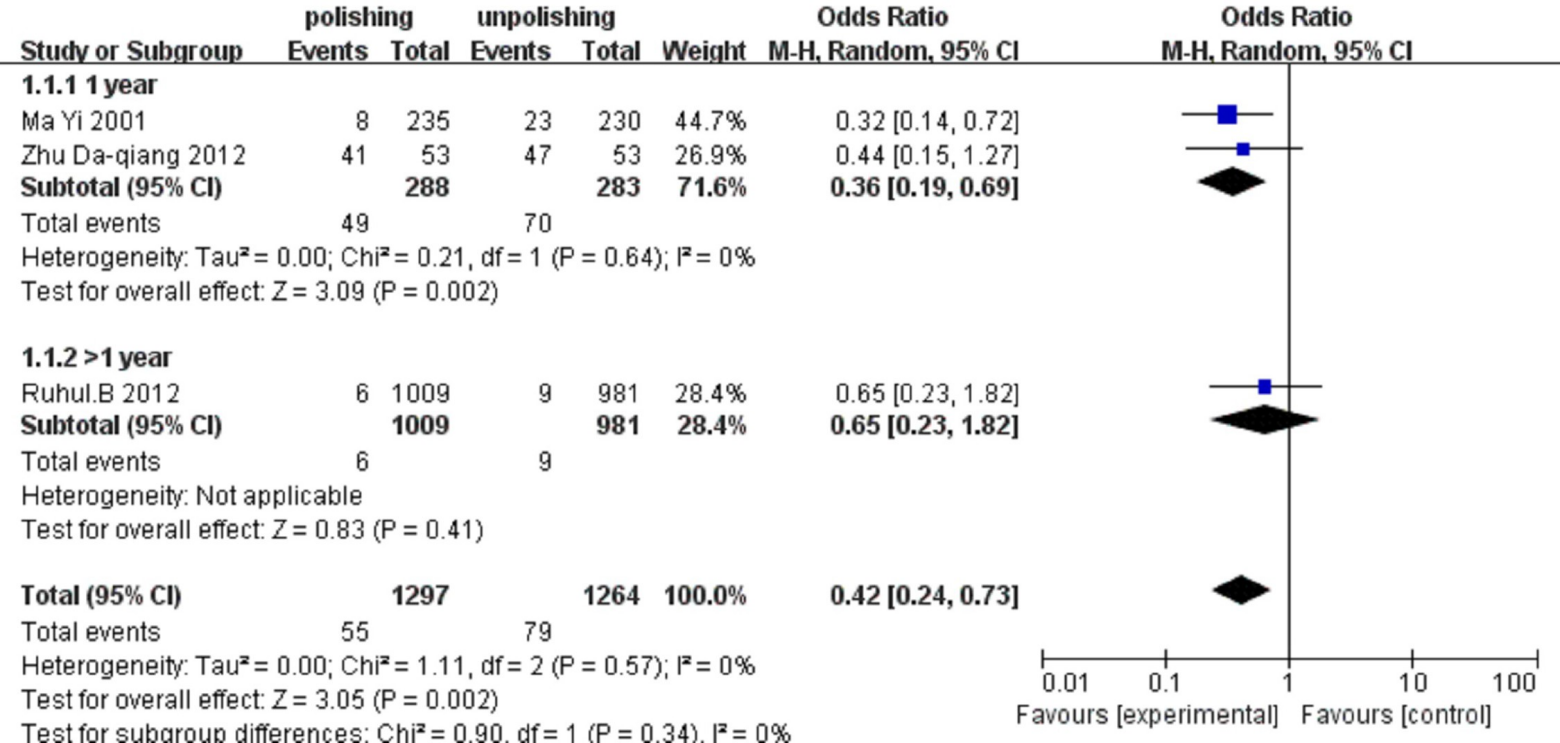
Comparing the occurrence of PCO between anterior capsule polish and unpolish groups after 1-year and longer follow-up. The invention of experiment group was anterior capsule polish; the control group was blank.

We defined the PCO as flaggy or schistose opacification appeared on the posterior capsule after phacoemulsification.[24] The PCO rates were reported in all 3 studies including 2561 eyes, and PCO rates were evaluated after at least 1 year in all eyes. The result about the effect of anterior capsule polish on PCO rate were showed in Fig 4. Summary odds ratio for the effects of polish on PCO rates was 0.42 (95% CI 0.24–0.73), suggesting that anterior capsule polish reduced the occurrence of PCO. However, some studies showed that there was no statistically signification difference between polish group and unpolish group in longer follow-up.[25]

### Publication bias

**Fig 5:**
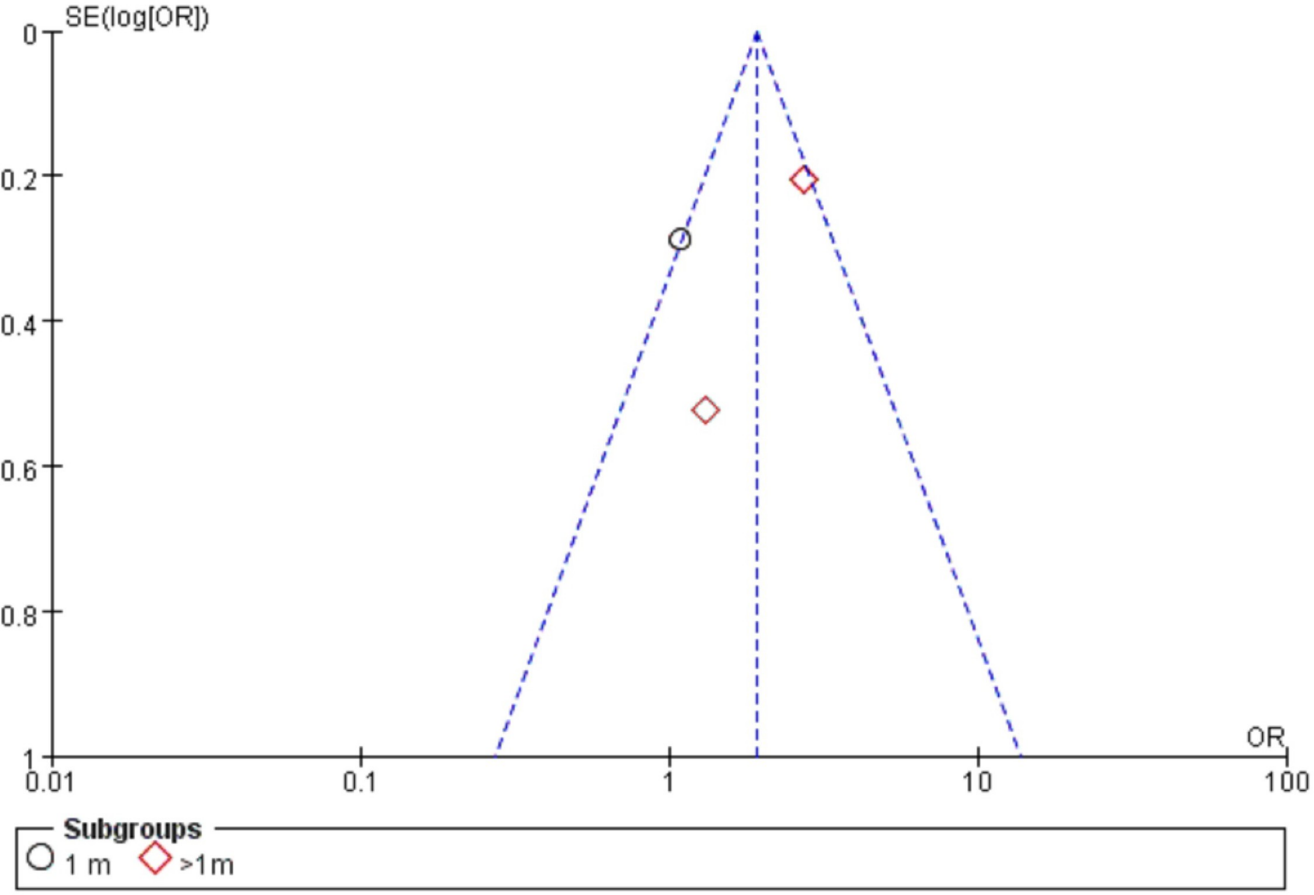
Funnel plot for the patient number of good UCVA for studies comparing anterior capsule polish group with anterior capsule unpolish group as a solo procedure.

Visual inspection of funnel plots by follow-up and patient number of good UCVA did not reveal any asymmetry Fig 5.

## Discussions

At present, cataract is the leading cause of blindness in the world and PCO remains the most common complication of modern cataract surgery. If the LECs were removed completely, PCO would be avoided possibly. There were controversial conclusions about anterior capsule polish in different studies. Therefore, a meta-analysis is required to evaluate the effects of anterior capsule polish on visual function. Our meta-analysis showed that the surgical method of polish was associated with visual function. The results of meta-analysis indicated that anterior capsule polish improved visual function.

In the meta-analysis, the PCO rates and visual acuity were performed as outcome variables based on the 5 included studies. Anterior capsule polish improved UCVA in 3-years follow-up period. The following factors are considered as reasons for good UCVA in polish group: anterior capsule opacification, capsule contraction, capsule stability, effective lens position.[16,26] The proliferation of LECs may lead to capsule contraction, which forces IOL to move forward and backward, resulting in refractive error. The corrected visual acuity using glasses may weak the effects of capsule contraction caused by LECs. Some studies indicated that anterior capsule polish would decreased anterior capsule opacification, which reduced the possibility of capsule contraction.[27] In addition, the IOL haptic design and material were reported to have effects on LECs proliferation and capsule contraction.[28,29] Both the implantation and location of IOL played important roles in anterior capsule stability and migration of LECs. After lens removed, the remnant LECs on the capsular bag underwent proliferation and transdifferentiation, resulting in capsule opacification. Anterior capsule opacification leads to refractive error. Some studies found that the material of IOL influenced on opacification rate by withholding migrating LECs on capsule.[30] The material and the design of IOL had effect on visual acuity after removing LECs on anterior capsule.

The migration of LECs on the posterior capsule leads to PCO. The presented study assessed the short-term influence of anterior capsule polish on PCO rates. The results of our meta-analysis found that incidence of PCO in polish group was less than that of unpolish group in short-term follow-up period. The longest follow-up among included studies was 3 years, indicating unclear effects of anterior capsule polish on longer follow-up period. A study showed that was no statistically significance different in 5-years follow-up between polish group and unpolish groups.[25] The difference might result from the unstable status of LECs in short follow-up. Anterior capsule polish enhanced remnant LECs growth in seven days postoperatively.[31] The LECs *in vivo* was presented in style of PCO, which was not directly obtained from human clinically. PCO impairs visual function after modern cataract surgery by reducing visual acuity and increasing intra-ocular straylight. Capsule opacification leads to scattered light.[7] The degree and the location of PCO both have effect on visual function impairment. Anterior capsule polishing would remove remnant LECs and reduce occurrence of PCO.

Many complications of modern cataract surgery are associated with the proliferation of LECs. Various means to destroy LECs, such as mitosis, heating, freezing, laser and trypsin, are not applicable.[32] In animal studies, LECs growth was inhibited with IOLs incubated in celecoxib.[33] Trypsin damages other structure in the anterior polar of eyeball. As a mechanical method, anterior capsule polish only touched LECs and was convenient to prevent complication of modern cataract surgery. A novel technique was designed to polish anterior capsule.[34] The polisher was used to remove remnant LECs at any point within capsular bag. The results of our meta-analysis suggested that anterior capsule polish is a kind of method to prevent the occurrence of PCO. One study stated that anterior capsule polish enhanced remnant LECs growth by stimulating remnant cell.[35] The proliferate ability of LECs after anterior capsule polish *in vivo* should be investigated in future.

## Strength and limitation

Many studies investigated the relationship between anterior capsule polish and PCO occurrence. Our meta-analysis focused on the effects of anterior capsule polish on visual function. In addition, our meta-analysis was performed in an academic medical setting according to PRISMA guidelines.

One limitation of our review was that 4 included studies were observational cohort studies. We hope that more RCTs about anterior capsule polishing will be performed in future. Another limitation was the short-term follow-up period. A longer follow-up period will help the clinicians better understand the surgical method of polish on visual function. The article number of our meta-analysis is also limited. Although we conducted a thorough electronic search and a manual search of the relevant publications, there were only 1 RCTs on the relationship between anterior capsule polish and visual function. More relevant RCTs would enrich our understanding on the effects of polish on visual function.

Anterior capsule polish prevents complication of modern cataract surgery and has good effects on visual function in short term follow-up period. Future studies are required to identify the relationship between anterior capsule polishing and refractive error.

## Conclusions and relevance

Anterior capsule polishing would prevent complication of modern cataract surgery and have good effect on visual function in short term follow-up period. Future studies are required to relationship between anterior capsule polishing and refractive error.

## Supporting information

S1 Checklist PRISMA-IPD checklist of items to include when reporting a systematic review and meta-analysis of individual participant data (IPD). (DOC)
